# Fas/Fas-Ligand Interaction As a Mechanism of Immune Homeostasis and β-Cell Cytotoxicity: Enforcement Rather Than Neutralization for Treatment of Type 1 Diabetes

**DOI:** 10.3389/fimmu.2017.00342

**Published:** 2017-03-27

**Authors:** Esma S. Yolcu, Haval Shirwan, Nadir Askenasy

**Affiliations:** ^1^Department of Microbiology and Immunology, Institute for Cellular Therapeutics, University of Louisville, Louisville, KY, USA; ^2^Frankel Laboratory of Experimental Bone Marrow Transplantation, Petach Tikva, Israel

**Keywords:** type 1 diabetes, β-cell inflammation, activation-induced cell death, Fas, Fas-ligand

## Introduction

Receptor/ligand interactions of the tumor necrosis factor (TNF) superfamily are associated with versatile apoptotic and regulatory signaling pathways in parenchymal tissues and the immunohematopoietic system. Apoptotic signaling mediated by Fas and TNF receptor-1 (TNF-R1) is one of the major cytotoxic mechanisms used by immune cells to kill endogenous cells and exogenous pathogens. In this capacity, these ubiquitous effector mechanisms of cell death, along with perforin/granzyme, are direct mediators of β-cell injury that is inflicted in autoimmune insulitis in type 1 diabetes (T1D). At the same time, the TNF family receptor/ligand interactions are prime constituents of immune homeostasis that enforce negative regulation of sensitized immunocytes (1–3). All immune cells upregulate TNF family receptors upon activation and are therefore submitted to negative regulation by apoptosis within the process of activation-induced cell death (AICD), which is also essential to termination of inflammation (4).

Tumor necrosis factor family receptor/ligand interactions participate as universal cytotoxic mechanisms in physiological turnover of cells, removal of dysfunctional cellular elements, and clearance of debris following injury of parenchymal tissues. As ubiquitous injury factors, the TNF family ligands concomitantly activate precursors and synergize with other growth factors to induce generation of renewed functional parenchymal cells to replace the injured tissue. For example, the Fas receptor and the cognate Fas-ligand (FasL) are constitutively expressed and transduce trophic regulatory signals in tissues such as glia, neurons, hepatocytes, and endothelium (5–7). Likewise, trophic signals are transduced by TNF superfamily receptors in hematopoietic stem and progenitor cells, which synergize with other growth factors to foster hematopoiesis under stress conditions (8). Therefore, common injury factors such as TNFα and interferons (IFN) couple hematopoietic activity to inflammation and injury in order to fuel immune reactions and support initial resolution of injury (9).

The Fas/FasL interaction is a common effector mechanism of β-cell apoptosis and islet injury under inflammatory conditions, and physiological modulation of immune homeostasis, including control of aberrant autoimmune reactions. In the absence of specific characteristics of the toxic and anti-inflammatory pathways, it is questioned whether neutralization or reinforcement of the Fas/FasL interaction is of therapeutic value in autoimmune disorders. We examine a wide array of evidence arguing in favor of and against therapeutic neutralization of Fas and/or FasL and conversely, consider the feasibility of implementation of this molecular interaction as approaches to abrogate autoimmune diabetes. Despite focus on a particular signaling pathway, the Fas/FasL interaction, in a particular autoimmune disorder, T1D, the debate is rather relevant to multiple receptor/ligand interactions of the TNF superfamily and to the entire range of inflammatory and autoimmune disorders.

## Why Focus on the Fas/FasL Interaction?

The Fas/FasL interaction attracts attention as the common executioner of apoptosis in the TNF superfamily endowed with distinct characteristics (10). Like perforin/granzyme (11, 12), apoptotic signaling by Fas/FasL is localized by the requirement of direct contact between the effector and target cells, a unique feature caused by essential Fas receptor trimerization through engagement of the membrane-bound or oligomers of the ligand. The physiological significance of this receptor/ligand interaction is emphasized by lymphoproliferative disorders resulting from disruption of homeostatic negative regulation in Fas-deficient (*lpr*) and FasL-defective (*gld*) mice (13). Despite these particular characteristics of the Fas/FasL interaction, common physiological trophic and apoptotic activities are shared by soluble ligands of the TNF superfamily, including TNFα and TNF-related apoptosis-inducing ligand receptor-1 (8).

## Physiological Activities of the Fas/FasL Interaction in Pancreatic Islets

### Non-Immunogenic Activities

Several components of the signaling pathways associated with the Fas receptor are involved in insulin secretion (14, 15) and physiological adjustment of β-cell mass. For example, the antiapoptotic factor FADD-like interleukin-1β-converting enzyme-inhibitory protein acting as a competitive caspase-8 antagonist promotes β-cell growth (16) under the inductive influence of interleukin-1β (IL-1β) (17). The Fas signaling pathway is also involved in regulation of insulin secretion (14, 15).

### Immunogenic Activities

Physiological immune privilege uses FasL to defend organs from excessive inflammation that is often more harmful than pathogens, such as the anterior eye chamber and reproductive organs (10, 18). The pancreas is not one of the first line immune privileged tissues. However, both islet cells and vascular endothelium constitutively express FasL, while the Fas receptor is prevalently detected in resident macrophages (19, 20) and in inflamed islets (21, 22). The histological pattern of expression is suggestive of a defensive rim of FasL-expressing α- and β-cells in the islets of Langerhans (23). In fact, the frontier battleground between reactive T cells and tissues is islet vasculature (24), which constitutively expresses both Fas and FasL (25) and is therefore inherently insensitive to Fas-mediated apoptosis (26, 27). Protection of the islets by relative immune privilege represents a wider network of defense of the pancreas from incidental inflammation and immune attack (28) due to the extensive digestive capacity of pancreatic enzymes responsible for intestinal degradation of substrates, which might cause autolytic pancreatitis associated with severe morbidity and mortality.

## The Fas/FasL Interaction in Inflammatory Insulitis

### The Fas/FasL Interaction As a Mechanism of β-Cell Death

Naïve islets express low levels of Fas (21, 22) and are intrinsically resistant to apoptosis triggered by this receptor (29, 30). Islets become gradually sensitive to apoptosis along the course of inflammation (31–33) due to increased Fas expression in β-cells (34, 35) and concomitant sensitization to Fas-mediated apoptosis (36, 37). Upregulated expression of the Fas receptor is one of the many features of the transcriptional profiles of inflamed islets (38, 39) caused by activation of the nuclear factor-κB (NFκB) pathways (40). Induced Fas transcription and modulation of expression is caused by a number of proinflammatory cytokines including IL-1α, IL-1β, IFNγ, nitric oxide (NO), and TNF-α that synergize with Fas as effector mechanisms of β-cell destruction (37, 41). Furthermore, both Fas expression induced by IL-1β (42) independent of NFκB activation (43, 44) and islet sensitization to apoptosis (45, 46) also evolve as direct consequences of hyperglycemia. The vast changes in transcriptomes and expression profiles of the inflamed islets may be viewed as an effort of the tissue to sustain insulin production and increase β-cell mass. However, cytokines secreted by the islets themselves paradoxally enhance immune activation and islet injury under inflammatory conditions (47–49).

### Potential Therapeutic Efficacy of Fas and/or FasL Neutralization

There is extensive evidence emphasizing a pivotal role of the Fas/FasL interaction in destructive insulitis in T1D, including experiments performed in transgenes deficient in the receptor and/or the ligand. For example, homozygous transgenes of non-obese diabetic (NOD) mice deficient in the Fas receptor (*lpr*) are protected from spontaneous evolution of diabetes, and heterozygous NOD.*lpr* transgenes display severe mononuclear infiltration in the islets without hyperglycemia (50, 51). However, NOD.*lpr* with and without superposed SCID mutations that display reduced incidence of spontaneous diabetes are susceptible to islet injury inflicted by adoptive transfer of diabetogenic cells (52). Likewise, disease incidence is reduced in FasL-deficient transgenes (*gld*) crossed onto the NOD background (52, 53). It is logical and tempting to approach inflammatory insulitis by neutralization of the Fas/FasL interaction as one of the pivotal cytotoxic mechanisms used by diabetogenic effectors to attack β-cells (54–57), similar to the potential therapeutic benefit of TNF-α neutralization (58). However, it should be noted that FasL neutralization in the early postnatal period (53, 59) and in very early stages of inflammation slowed the pace but failed to prevent evolution of insulitis (60).

## Arguments Against Therapeutic Neutralization of the Fas/FasL Interaction

### Multiple Immune Mechanisms Modulate Inflammatory Insulitis in the Absence of Functional Fas/FasL Signaling

The insights into effective disease diversion by interruption of functional Fas/FasL signaling led to identification of a number of indirect immunogenic factors that modulate the course of inflammation in transgenic mice. The variable patterns of disease expression in *lpr* transgenes are explained by two mechanisms beyond relative insensitivity of Fas-deficient islets to apoptosis. First, slow pace of spontaneous inflammatory insulitis is attributed to reduced aggressiveness and slow proliferation of effector lymphocytes of the *lpr* transgenes (61). Second, T cells of *lpr* transgenes overexpress FasL, which inhibits the activity of endogenous and adoptively transferred diabetogenic cells (62). In variance, the pace of disease is slowed in *gld* transgenes by the activity of B lymphocytes that attenuate the course of inflammation by enhanced secretion of IL-10 (63). It is therefore evident that disruption of a pivotal immune homeostatic mechanism such as the Fas/FasL interaction has quite significant consequences that affect autoimmunity beyond direct participation as a cytotoxic mechanism of islet injury.

### Multiple Redundant Mechanisms of β-Cell Death Obviate Fas Neutralization

The difficulty in designation of an exact role of Fas-mediated apoptosis stands in the multiple, redundant, and interrelated mechanisms of β-cell death in T1D. The progressive involvement of Fas as a mediator of apoptosis along the course of inflammation has been challenged by a series of studies showing that this mechanism is neither obligatory nor essential in the process of destructive insulitis (64–68). Although Fas expression correlates with β-cell inflammation and unequivocally contributes to destructive insulitis, a causal relationship is rather complex because apoptosis also correlates with upregulation of granzyme and TNF-R1 (69–72). In addition to the canonical mechanisms of apoptosis, islet injury is inflicted by a number of cytotoxic cytokines such as IL-1β, IFNγ, and NO (73, 74). It is quite difficult to attribute distinct activities to these interrelated mechanisms in the process of inflammatory insulitis, because most cytokines as well as TNFα induce Fas expression (21, 22, 29, 75).

### Individual Cytotoxic Mechanisms Are Dispensable in Islet Destruction

Each one of the canonical cytotoxic mechanisms, including Fas, TNF-R1, and perforin/granzyme, is dispensable in autoimmune β-cell destruction (68, 75–80), as well as islet allograft rejection (27, 81). Outstanding is the compensation of dysfunctional Fas/FasL interactions by other effector mechanisms of β-cell death (11, 35, 69, 82–84). Likewise, redundant activity of TGF-β, IL-1β, IFNγ, and NO is a common characteristic, as each individual cytotoxic mechanism is largely dispensable in β-cell lysis (36, 41, 47, 85).

### Harnessing Physiological Mechanisms to Counteract Islet Inflammation

Extending the mechanism of immune privilege, negative regulation of immune cells by TNF family receptor/ligand interactions has significant homeostatic impact on the intensity of inflammatory reactions. The common mechanism of tissue defense involves induction of apoptosis in autoreactive effectors sensitive to AICD at the level of pancreatic islets *in situ* (30, 62, 86). Physical elimination of diabetogenic cells has been attained by targeted expression of TNFα (87–90) and TGFβ (91, 92) under control of the insulin promoter, systemic administration of TNF-α (93–95), and overexpression of FasL protein in regulatory T cells (30, 96).

### Therapeutic Implications of the Fas/FasL Interaction

The analysis presented here suggests that inhibition of the Fas/FasL interaction has little potential efficacy in prevention of β-cell destruction by inflammatory insulitis, while targeted reinforcement of this mechanisms of immune homeostasis holds the potential to abrogate diabetic autoimmunity. Signaling through Fas receptor is one of many redundant and dispensable mechanisms of β-cell lysis by autoimmune attack; thus, neutralization provides transient symptomatic relief with little impact on alternative cytotoxic mechanisms (64–67). Implementation of FasL neutralization for treatment of T1D might even result in increased incidence of malignancies as seen with TNF-α inhibitors (97–99). On the contrary, the role of FasL along TNF-α and perforin/granzyme is much more significant and non-redundant in immune homeostasis than in induction of β-cell death (10, 61, 62, 88, 100). These homeostatic immune mechanisms counteract inflammation as mediators of effector cell death (27, 30, 86, 89–96) are mandatory to reinstitution of suppressor mechanisms (101) and are indispensable in termination of inflammatory reactions (4).

## Author Contributions

All authors contributed equally to this manuscript.

## Conflict of Interest Statement

HS and EY are inventors of several patents related to Fas-ligand. NA discloses no potential conflict of interest.
